# Assessing the Diagnostic Performance of a Smart Bra Using Temperature and Bioimpedance for Breast Cancer Detection: A First-in-Human Study

**DOI:** 10.3390/s26092869

**Published:** 2026-05-04

**Authors:** Anne-Sophie Belmont, Marie-Valérie Moreno, Eloise Aubret, Justine Dubreuil, Nathalie Piazon, Julien Berthiller, Maxime Bonjour, Emmanuelle Dantony, Audrey Haquin, Marion Cortet

**Affiliations:** 1 Plateforme Transversale de Recherche Clinique de l’Institut de Cancérologie, Hospices Civils de Lyon, 69495 Pierre-Bénite, France; 2 Research and Development Center, RunSys, 162 Lot le Verger, 69380 Chasselay, France; 3 Service de Gynécologie, Hôpital de la Croix-Rousse, Hospices Civils de Lyon, 69004 Lyon, Francemarion.cortet@chu-lyon.fr (M.C.); 4 Pôle de Santé Public, Hospices Civils de Lyon, 69 003 Lyon, France; 5 Service d’Imageries Médicales, Hôpital de la Croix-Rousse, Hospices Civils de Lyon, 69004 Lyon, France

**Keywords:** breast cancer screening, wearable medical device, thermography, bioimpedance, smart bra, first-in-human study

## Abstract

(1) Background: Breast cancer screening remains limited by mammography, particularly in younger women, in dense breast tissue, and in the detection of interval cancers. The PHI-BRA Smart Bra was developed as a wearable, non-invasive device combining thermography and bioimpedance for frequent breast monitoring. This first-in-human study aimed to assess the feasibility and in vivo diagnostic performance of the PHI-BRA system in discriminating between women with and without breast lesions. (2) Methods: A prospective feasibility study was conducted between March 2023 and February 2024. A calibration cohort (*n* = 15) was used to define the discrimination model, followed by an analysis cohort (*n* = 26; 13 with breast lesions and 13 without). Thermal and bioimpedance signals were acquired using the PHI-BRA device. Diagnostic performance was evaluated using receiver operating characteristic (ROC) analysis, with mammography as the reference standard. (3) Results: In the analysis cohort, the temperature-based model achieved an area under the ROC curve (AUC) of 80.8% (95% CI [63.2–98.3]). At the optimal threshold, sensitivity was 84.6% (95% CI [61.5–100]) and specificity was 76.9% (95% CI [53.8–100]). Exploratory bioimpedance analyses showed lower sensitivity but high specificity, mainly limited by sensor contact stability. No adverse events were reported. (4) Conclusions: This first-in-human study demonstrates an initial exploration of the feasibility and safety of a wearable thermography-based approach for breast lesion discrimination. The results support further clinical validation of a multimodal wearable system as a complementary tool to existing breast cancer screening strategies.

## 1. Introduction

Breast cancer remains a major public health concern, with its incidence continuing to rise worldwide. It is the second-most commonly diagnosed cancer globally, accounting for 2.3 million new cases in 2022, and is the fourth leading cause of cancer-related deaths across all sexes [1].

Current screening methods rely primarily on mammography. However, mammography exposes patients to ionizing radiation, which accumulates with repeated screenings over time. Additionally, its sensitivity decreases in the presence of dense breast tissue, particularly in premenopausal women. As a result, mammography is not ideally suited for screening younger women, a population in which breast cancer incidence has been increasing in recent years. Another major challenge is the issue of interval cancers, which are cancers diagnosed between two regular screenings. The rate of interval cancers is estimated at 18% [2]. Increasing the frequency of mammography (e.g., annual screening) is not a viable solution, as it would raise both the risk of false positives and cumulative radiation exposure.

These limitations have driven research into alternative breast cancer screening approaches that are safe, accessible, and suitable for frequent use [3]. Over the past 40 years, various technologies have been explored, including thermography [4], microwave imaging, electrical impedance tomography (EIT), bioimpedance spectroscopy (BIS), and pressure-sensing devices, aiming to provide adjunct screening options and expand the tools available to healthcare providers and patients [3,4,5].

However, despite decades of research, these technologies still face critical challenges, particularly in terms of sensitivity, specificity, and limited large-scale clinical validation.

For example, thermography has received FDA clearance as an adjunctive diagnostic tool for breast cancer, but it is not recommended as a primary screening or diagnostic method due to its low sensitivity and specificity [6,7]. Electrical impedance spectroscopy (EIS) has also been widely investigated as an inexpensive and non-invasive screening technique, with reported sensitivities of up to 82.62% and specificities of up to 95.79% [8,9,10,11].

In 2019, Moreno et al. [2] proposed combining thermography and EIS into a wearable device—a Smart Bra (PHI-BRA)—to improve diagnostic reliability. Their preliminary study on breast phantoms showed promising results, with a statistically significant difference in the discrimination between “healthy” and “tumor” phantoms (*p* = 0.008, Student’s *t*-test; Shapiro–Wilk *p* = 0.846, indicating normal distribution; Fisher’s variance test *p* = 0.287).

The objective of the present paper is to report the first in vivo diagnostic performances of the PHI-BRA Smart Bra, assessing its ability to discriminate between participants with and without breast lesions in a first-in-human feasibility study.

## 2. Materials and Methods

A prospective feasibility study was conducted between March 2023 and February 2024 to evaluate the diagnostic performances of the PHI-BRA device and compare it to the current gold standard, mammography. This study was not intended for CE marking purposes.

Ethical approval was obtained from the French National Agency for Medicines and Health Products Safety (ANSM) on 28 September 2022, and approval from the Ethics Committee (CPP) was granted under the ID-RCB number 2022-A01524-39 on 22 August 2022. The ClinicalTrials.gov identifier is NCT05574816. The first patient was enrolled on 30 March 2023.

### 2.1. Methodology

The Smart Bra relies on thermal and bioimpedance measurements. These measurements, collected at different points on the breast, are used to build a model that generates a single value. This value serves to classify patients into two groups: those without breast lesions and those with breast lesions. Accordingly, the study was divided into two phases. The calibration cohort comprised fifteen participants: seven “with breast lesion” and eight “without breast lesion”, to define the optimal discrimination model. The analysis cohort comprised 26 participants: 13 “with breast lesion” and 13 “without breast lesion”, to blindly assess the device’s diagnostic performances using the model defined in phase one.

Participants were recruited at Lyon’s University Hospital. Those “without breast lesion” (classified as ACR1 or ACR2 after mammography) were recruited from the radiology department, either on the same day as their imaging or within one month, depending on their availability. Those “with breast lesion” (classified as ACR4 or ACR5) were recruited from the gynecology department between biopsy and surgical intervention.

Inclusion criteria for both groups: women aged 18 to 85 years; no prior breast cancer surgery (lumpectomy, mastectomy); bra size corresponding to S/M or L/XL (cups B to E); ability to understand the information sheet; signed informed consent; affiliation with the French national healthcare system.

Specific criteria for the “with breast lesion” group: lesions classified as ACR4b, ACR4c, or ACR5 confirmed by core needle biopsy; no superficial hematoma following biopsy.

Specific criteria for the “without breast lesion” group: negative mammography (ACR 1 or 2).

Non-inclusion criteria for both groups: personal history of breast cancer (due to glandular distortion from surgery and/or radiotherapy); ACR3 or ACR4a lesions, pregnant or breastfeeding women; presence of a pacemaker or breast implants; known allergy to device materials; under legal protection or deprived of liberty; receiving psychiatric care or institutionalized for non-research purposes.

Device set-up and measurements were performed by trained clinical research associates (CRAs). For “with breast lesion” participants, measurements were performed in the gynecology department, and for “without breast lesion” participants, they were performed in the radiology department. All measurements were conducted in the same dedicated room, though ambient temperature was not controlled (the rooms were equipped with thermostats to regulate temperature regardless of the season, and the device was placed several minutes before measurement to ensure thermal equilibrium between the skin and the electrodes). For “with breast lesion” participants, the device was applied to the affected breast. For “without breast lesion” participants, the laterality of the studied breast was randomized. Bra size determined the device size (S/M for cups B-C and L/XL for D-E). The device was centered on the nipple and secured using a sport bra. Participants remained in a semi-seated position for approximately 10 min during measurements. Incomplete measurements led to participant replacement.

### 2.2. Materials

**Figure 1:**
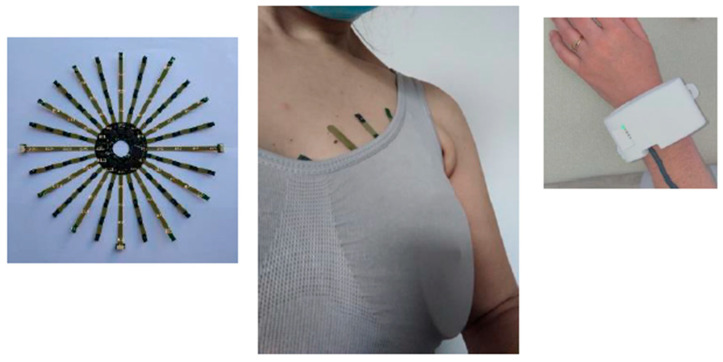
Photograph of the PHI-BRA sensor centered around the nipple and held in place by a sport bra. The data acquisition unit is placed on the participant’s wrist.

The PHI-BRA device system includes a patented system [12]. A “micro” unit in Protogen 18420 (USP Class VI biocompatible), housing a BLE communication module (SPBTLE-RF0TR de chez STMicroelectronics) and a battery (lithium-polymer LP-503759-1S-3 from BAK, with a voltage of 3.7 V). The system allows continuous operation for eight days without recharging (to facilitate the work of medical staff). The sensor has a capacity of 13,500 mA/h, providing a margin of more than 25% compared to the 960 mA/h calculated based on the measurement protocol;

- A 10-wire twisted cable compliant with ISO 10993-5 [13]
- A flexible “sensor cap” board (PCB covered with biocompatible coverlay) with stainless steel electrodes (316 L (EN 1.4435), obtained by laser cutting, then polished and cleaned according to the procedure AMDEC-IQ-PQ-OQ), attached using a silver-conductive two-part adhesive; and a tablet controlling the software and storing data (Figure 1).

The sensor cap includes 24 branches: 12 branches with five temperature sensors (MAX30205MTA+T from Analogic Device) each (60 total) and 12 branches with five multiplexed (ADG731 from Analogic Device) electrodes connected to a bioimpedance sensor (60 total). The device comes in two sensor cups.

The geometry was defined to scan the entire breast with the best possible resolution using multiplexing and to be able to produce bioimpedance images.

A bonnet card size L/XL is indicated on a label positioned on the central component. The central part of the bonnet card is a disk with a diameter of 64 mm, pierced in its center by a hole with a diameter of 19.7 mm. The arms are 5 mm wide, and the longest arm is 91.3 mm. A bonnet card size S/M is indicated on a label positioned on the central component. Only the arm lengths differ, with the longest arm measuring 73 mm.

To ensure EMC (electromagnetic compatibility), 1 kΩ resistors were placed at the ends of the traces. Furthermore, analog data are converted within the sensor board and then transferred digitally from the sensor board to the microcontroller board.

Three frequencies were used for bioimpedance measurements: 4 kHz to explore extracellular matrix, 40 kHz to explore membrane permeability and 80 kHz to explore intracellular matrix. For each frequency, resistive (R, in ohms) and capacitive (X, in ohms) values were recorded. The system accuracy was tested and evaluated on universal phantom for temperature measurement (repeatability error < 1%, accuracy error < 1.5%) and for bioimpedance measurement (repeatability error < 1%, accuracy (R) = 1.6%, accuracy (X) = 1.8%, on R (680 Ohm)//R (910 ohm) C (2.7 nF) bench).

### 2.3. Data Analysis

Calibration Cohort: Data were analyzed by Runsys. Participants were divided into a training set (eight participants (four with lesions, four without)) and a validation set (seven participants (three with lesions, four without)). Multiple algorithmic approaches were tested, including binary logistic regression models, partial least-square models and principal component regression models, using temperature, bioimpedance, and a combination of temperature and bioimpedance. The choice was made based on models offering the best results while limiting the number of false positives and false negatives. Models were generated using XLStat. A 0.5 (50%) classification threshold was used. If the diagnostic score obtained through the discrimination model was over 0.5, the patient was classified as “positive” for breast lesions; if the diagnostic score was under 0.5, the patient was classified as “negative” for breast lesions.

Analysis Cohort: One model (binary logistic law (Equation (1)) was selected by Runsys to blindly assess the diagnostic score of the 26 analysable participants included in the analysis cohort.

Prédiction (lesion) = (1 −(1/(1 + EXP(−(a − b × Var))))) × 100(1)

A reduction in variables was carried out in order to improve the interpretability of the model and to limit noise. The average temperatures of the breast was chosen instead of the temperature at each point.

In the case of the bioimpedance model (partial regression law of linear least-squares), the reactance at 80 kHz was not retained in order to avoid generating multicollinearity with the reactance at 40 kHz.

The choice was made to retain the reactance at 4 kHz despite its bias due to contact impedance, because of the interest of this frequency for exploring information on the permeability of the cell membrane, which is hypothetically impacted by inflammation induced by lesions. Overfitting was primarily controlled by separating the data into training and validation groups. The limitation of the number of variables relative to the sample size was also respected, and the variables retained were the resistance and reactance at 4 and 40 kHz, and the resistance at 80 kHz.

These results were merged with the e-CRF collected data and sent to the Lyon University’s Hospital Biostatistics and Bioinformatics Department. Statistical analyses were performed using R (version 4.2.2) [14] and the pROC library. No data imputation was applied. Descriptive statistics were used for quantitative variables, while frequency tables were generated for qualitative data. Two-sided 95% confidence intervals (95% CIs) were computed for all statistical tests. Receiver operating characteristic (ROC) curves and corresponding areas under the curve (AUCs) were calculated from the diagnostic scores (reference standard: mammography) using DeLong’s method, with 95% CIs (primary endpoint). The optimal threshold was determined using the Youden Index, and its associated sensitivity and specificity were reported. The corresponding 95% CIs were determined using bootstrap methods. The association between temperature values and tumor volume was assessed using Spearman’s rank correlation (secondary endpoints).

## 3. Results

### 3.1. Calibration Cohort

**Figure A1:**
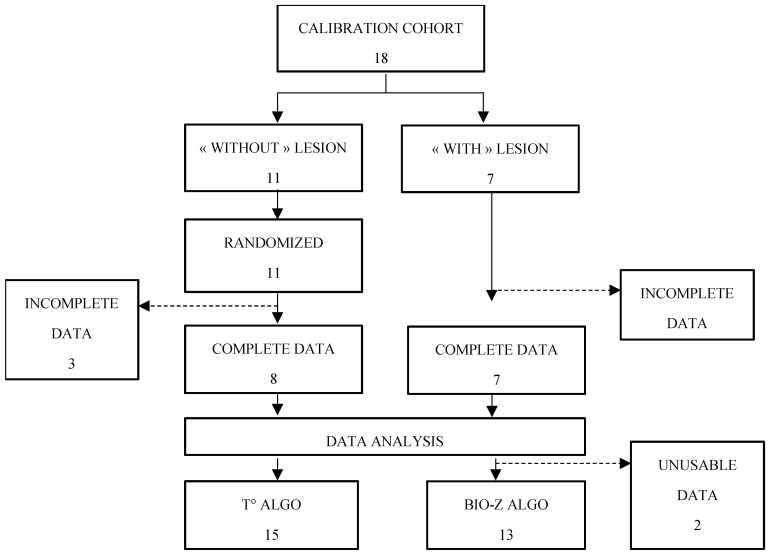
Calibration cohort participant flow chart.

Out of eighteen participants enrolled, three were excluded due to device failure (Figure A1: participant flow chart). The failure was traced to a firmware delay and was resolved by implementing a watchdog timer. No adverse effect (AE) or severe adverse effect (SAE) was reported.

Three binary logistic regressions were tested using temperature data, six partial least-squares regressions using bioimpedance data, and six principal component regression (PCR) using a combination of temperature and bioimpedance data. A model was selected for each category to determine the participants’ diagnostic scores. At a 0.5 threshold, the results included one false positive for the temperature model, one false positive and one false negative for the bioimpedance model, and hundred percent correct predictions for the model combining temperature and bioimpedance. Results are presented in Table A1.

Only one set of diagnostic scores was planned for analysis by the statistical department to assess the PHI-BRA device’s diagnostic performance as the primary criteria of the trial. Due to contact stability concerns, a conservative decision was made to use the temperature model, as instability of the bioimpedance sensors could have impacted the assessment of the device’s performance.

### 3.2. Analysis Cohort

**Figure 2:**
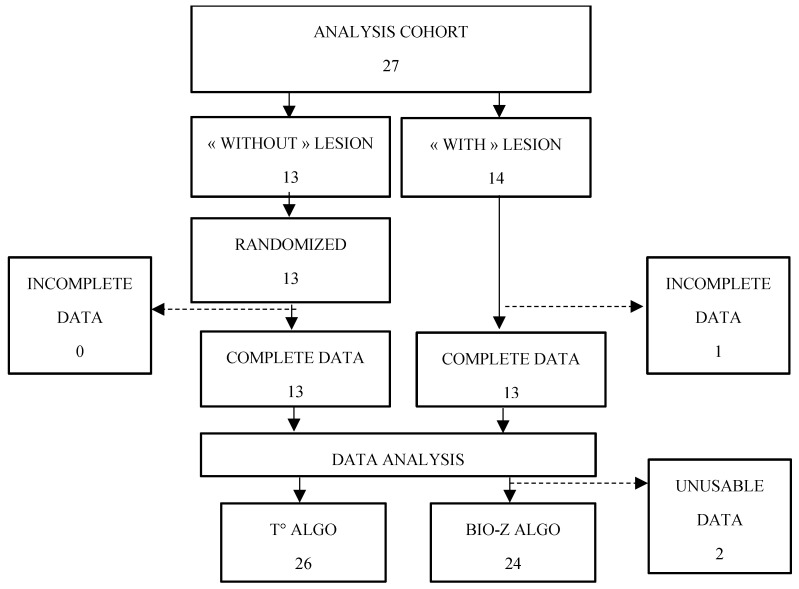
Analysis cohort participant flow chart.

No AE or SAE was reported across the 27 enrolled participants, confirming a favorable safety profile. Out of 27 participants enrolled, one was excluded due to incomplete measurement (Figure 2). Patient characteristics are in Table 1. The mean diagnostic score predicted by the temperature model for the “with breast lesion” group was 0.661 (66.1%; SD = 21.2), and it was 0.382 (38.2%; SD = 23.3) for the “without breast lesion” group.

**Figure 3:**
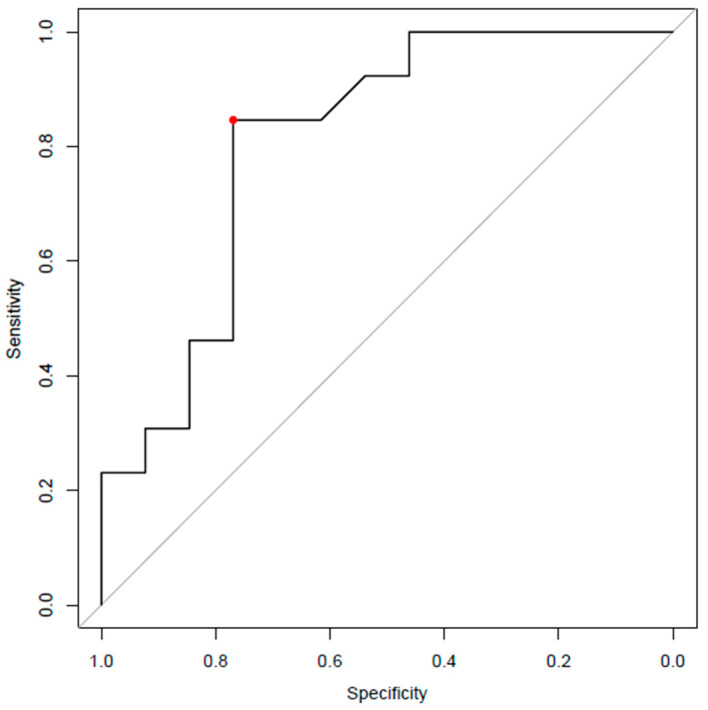
ROC curve using scores provided by the temperature model. The red point corresponds to the Youden Index, where sensitivity and specificity are maximal.

The area under the ROC curve (AUC), assessed using scores provided by the temperature model, is 80.8% (95% CI [63.2–98.3]) (Figure 3). The optimal threshold (0.458), determined by the Youden Index, yields a sensitivity of 84.6% (95% CI [61.5–100]) and a specificity of 76.9% (95% CI [53.8–100]).

**Figure A2:**
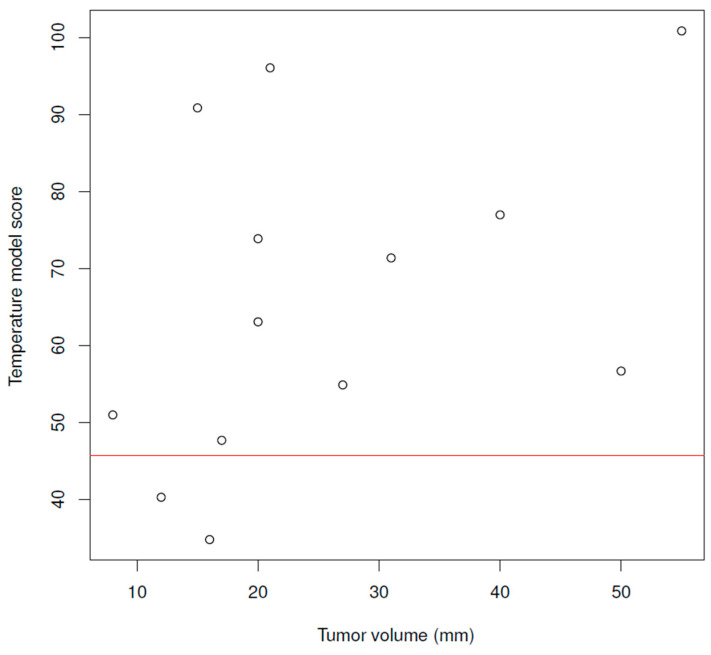
Spearman correlation coefficient using temperature model diagnostic scoring.

A moderate positive correlation was observed between tumor volume and measured temperature (Spearman’s ρ = 0.545) (Figure A2).

In addition to these trial criteria endpoints analyses, exploratory analyses were conducted to assess the PHI-BRA diagnostic performances, using scores provided by the bioimpedance model. This score was available for 24 patients. The AUC was calculated at 61.8% (IC 95% (38.1; 85.5). The optimal threshold (0.62), determined by the Youden Index, yields a sensitivity of 41.7% (95% CI [16.7–66.7]) and a specificity of 91.7% (95% CI [75–100]).

Exploratory analyses were not conducted to assess the PHI-BRA diagnostic performance using scores provided by the combination of the temperature and bioimpedance model due to the moderate performance of the bioimpedance model.

## 4. Discussion

Despite the very encouraging results obtained with the binary logistic regression temperature model (sensitivity: 84.6%, 95% CI [61.5–100]; specificity: 76.9%, 95% CI [53.8–100]) using the optimal threshold of 0.458, these findings must be interpreted with caution. Thermography, which detects abnormal vascular activity often associated with malignancies due to the higher metabolic rate and denser capillary network of cancerous tissues [15], still lacks validation in real-world clinical settings [7], despite recent advancements in texture analysis and machine learning approaches [16]. Its diagnostic reliability remains controversial, with reported sensitivity ranging from 25% to 97% and specificity from 12% to 85% [6,17]. Benign conditions such as mastitis, hormonal fluctuations, or dermatitis may mimic malignant thermal signatures, while slow-growing or deeply located tumors can lead to false negatives. In addition, temperature measurements were not performed under strictly standardized environmental conditions, as data acquisition occurred in different rooms across the two cohorts, potentially introducing variability related to ambient temperature or skin acclimatization. Furthermore, the optimal classification threshold was derived directly from the study population using the Youden index, which may lead to overoptimistic performance estimates. Consequently, external validation under standardized acquisition conditions—ideally including dedicated sensors for ambient and skin temperature monitoring—will be required to confirm the reproducibility and clinical applicability of these findings.

Importantly, beyond these limitations, this exploratory study successfully demonstrates the technical feasibility of temperature-based signal acquisition using the PHI-BRA system in a real-world clinical environment and provides a coherent physiological signal consistent with known mechanisms of breast cancer development. These results therefore represent a meaningful first step, supporting the relevance of the proposed approach and justifying further confirmatory investigations.

Bioimpedance analysis was explored as a secondary modality and was not included in the predefined primary endpoints of the study. Accordingly, only one algorithmic model—based on temperature-derived features—was formally trained and statistically validated, while bioimpedance data were assessed in an exploratory manner. Bioimpedance remains of strong interest, as it probes intrinsic electrical properties of breast tissue directly related to tissue composition and cellular architecture and is theoretically less sensitive to external physiological or environmental confounders than thermography. In the present study, bioimpedance measurements showed limited diagnostic performance, primarily due to signal instability associated with electrode–tissue contact, particularly at low frequencies. When benchmarked against previously published data, the observed performance falls within the lower range reported for in vivo bioimpedance-based techniques, with sensitivities around 75% and specificities between 72% and 82% under controlled acquisition conditions [18,19]. Direct comparison across studies remains challenging; however, as reported performances strongly depend on experimental settings, signal processing pipelines, and clinical objectives. Higher accuracies described in ex vivo studies [20] or in prognostic contexts [21] are therefore not directly comparable to the present work. Several authors [22] have highlighted the role of potassium channels in the communication between cancer cells and their microenvironment, thereby promoting tumor growth from the earliest stages of malignant transformation. Other studies have strengthened this hypothesis by demonstrating the involvement of cationic TRP channels in breast cancer [23]. These alterations in the expression and distribution of ion channels within tumor cell membranes modify their permeability, a parameter that can be assessed through the imaginary component of complex impedance, namely reactance. These relatively recent findings further support the relevance of pursuing the development of a bioimpedance measurement chain in order to enhance the diagnostic value of the proposed solution.

Beyond modality-specific considerations, several study design limitations must be acknowledged. For practical reasons, the initially planned healthy-to-sick participant ratio (1:5) was adjusted to 1:1 to facilitate recruitment within funding constraints. While appropriate for an exploratory study, this ratio does not reflect real-world disease prevalence and precludes reliable estimation of predictive values. Future studies should therefore adopt prevalence-adjusted cohorts. In addition, given the known variability in mammography sensitivity across age groups, subgroup analyses and the inclusion of diverse lesion types will be essential to better position the device within existing diagnostic pathways. The small sample size (*n* = 26), the single-center design, and the absence of inter-operator or inter-session reproducibility assessment further limit generalizability. These aspects will need to be addressed through multicentric studies incorporating repeated measurements to evaluate robustness across operators and clinical environments.

## 5. Conclusions

Taken together, these findings support a multimodal diagnostic perspective. Thermography and bioimpedance capture distinct but complementary physiological information—vascular and metabolic activity on the one hand, and tissue structural properties on the other. Within such a framework, thermography may serve as a sensitive screening component, while bioimpedance could contribute to improving specificity once technical robustness and standardized acquisition protocols are achieved. Although diagnostic precision remains a central objective, the repeatability of the PHI-BRA system may ultimately represent one of its key strengths, enabling longitudinal monitoring and early detection, particularly between routine imaging examinations or in higher-risk populations. Further longitudinal trials with larger datasets, potentially leveraging artificial intelligence to integrate clinical and contextual parameters, will be required to confirm these exploratory findings and refine these preliminary diagnostic algorithms [24]. A new longitudinal study on larger samples will be conducted after in-depth work on the prototype. Contact will be quantified using bipolar or tripolar measurements before launching quadripolar measurements. To improve reproducibility, various anatomical alignment features will be added, along with pressure quantification, which will influence contact.

## Figures and Tables

**Table 1:** Analysis cohort characteristics.

	“With Breast Lesion” (*n* = 13)				“Without Breast Lesion” (*n* = 13)			
Age	58.170 (SD 9.51)				60.727 (SD 8.637)			
Menopause Status	9 (Yes)	4 (No)			10 (Yes)	3 (No)		
Cup Size	4 (B)	5 (C)	2 (D)	2 (E)	6 (B)	3 (C)	4 (D)	0 (E)
Breast Size	1 (85)	3 (90)	6 (95)	3 (100)	3 (85)	4 (90)	4 (95)	2 (100)
ACR Status	0 (1 or 2)	9 (4)	4 (5)		13 (1 or 2)	0 (4)	0 (5)	

## Data Availability

The original contributions presented in this study are included in the article. Further inquiries can be directed to the corresponding authors.

## Appendix A

**Table A1:** Diagnostic scores assessed using three different models (temperature, bioimpedance and a combination of temperature and bioimpedance) to discriminate participants with and without lesions at a 0.5 threshold (scores < 0.5 qualified as “without lesion, scores > 0.5 qualified as “with lesion”). Dataset: calibration cohort participants used to train and validate the models. False positives (FP) and false negatives (FP) are in bold.

Set	Reference (Mammography Assessment)	Score Using T°	Score Using Bioimpedance	Score Using T° and Bioimpedance
Training	Without	0	ND	ND
Training	Without	0	0.21	0.18
Training	Without	0	0.31	0.20
Training	Without	0	0.19	0.22
Training	With	1	0.56	0.64
Training	With	1	**0.40**	0.74
Training	With	1	0.55	0.77
Training	With	1	0.99	1.16
Validation	Without	0.40	**0.62**	0.19
Validation	Without	0.00	ND	ND
Validation	Without	0.00	0.35	0.03
Validation	Without	**1.00**	0.04	0.19
Validation	With	0.99	1.34	0.96
Validation	With	0.97	0.64	1.01
Validation	With	1.00	0.80	0.86
